# Can Crop Models Identify Critical Gaps in Genetics, Environment, and Management Interactions?

**DOI:** 10.3389/fpls.2020.00737

**Published:** 2020-06-12

**Authors:** Claudio O. Stöckle, Armen R. Kemanian

**Affiliations:** ^1^Department of Biological Systems Engineering, Washington State University, Pullman, WA, United States; ^2^Department of Plant Science, The Pennsylvania State University, University Park, PA, United States

**Keywords:** improvement, crop models, uncertainty, GxExM, simulation performance

## Abstract

Increasing food demand under climate change constraints may challenge and strain agricultural systems. The use of crop models to assess genotypes performance across diverse target environments and management practices, i.e., the genetic × environment × management interaction (GEMI), can help understand suitability of genotype and agronomic practices, and possibly accelerate turnaround in plant breeding programs. However, the readiness of models to support these tasks can be debated. In this article, we point out modeling and data limitations and argue the need for evaluation and improvement of relevant process algorithms as well as model convergence. Under conditions suitable for plant growth, without meteorological extremes or soil limitation to root exploration, models can simulate resource capture, growth, and yield with relative ease. As stresses accumulate, the plant species‐ and genotype-specific attributes and their interactions with the soil and atmospheric environment generate a large range of responses, including conditions where resources become so limiting as to make yields very low. The space in between high and low yields is where most rainfed production occurs, and where the current model and user skill at representing GEMI varies. We also review studies comparing the performance of a large number of crop models and the lessons learned. The overall message is that improvement of models appears as a necessary condition for progress, and perhaps relevancy. Model ensembles help mitigate data input, model, and user-driven uncertainty for some but not all applications, sometimes at a very high cost. Successful model-based assessment of GEMI not only requires better crop models and knowledgeable users, but also a realistic representation of the environmental conditions of the landscape where crops are grown, which is not trivial given the 3D nature of water and nutrient transport. Models remain the best quantitative repository of our knowledge on crop functioning; they contain a narrative of plant, soil, and atmospheric functioning in computer language and train the mind to couple processes. But in our quest to tame GEMI, will they lead the way or just ride along history?

## Introduction

Increasing demand for higher quality and quantity of food under a changing climate with more frequent and severe heat, drought, and flood events poses a significant challenge for agriculture. It is also expected that agriculture meets this increasing demand while polluting less. The use of crop models to assess genotypes performance across diverse target environments and management practices, i.e., the genetic × environment × management interaction (GEMI), can help understanding genotype suitability, best agronomic management, and possibly provide a valuable tool for fast-turnaround in plant breeding programs. This paper is concerned with the role of crop models in assessing GEMI. Our perspective is from the viewpoint of crop model development and their adaptation for current and emerging applications. These applications can be divided in different types, for example those attending breeding program needs and pertaining to field and landscape management. Ideally, there should be no boundary between these applications, but research teams have had and still have different missions that make for diverging modeling strengths.

Process-based crop models integrate mathematical descriptions of the mechanisms leading to growth and yield of crops in response to environmental and management conditions. Through the twentieth century, the experimental and conceptual understanding of main processes allowing quantitative descriptions of crop growth advanced steadily. With the advent of personal computers in the early 1980s, these processes were integrated as concise algorithms in crop simulation models able to deal with some aspects of GEMI. These modeling systems keep evolving, integrating crop rotations, tillage, soil carbon, and other nutrients cycling. Advances in database management, spatial analysis tools, and cluster and cloud computing are creating new opportunities for model development and applications.

For decades, crop simulation models have been touted as tools with potential to evaluate crop genotype responses to changes in the environment and management (O’Toole and Stockle, 1991; Boote et al., 2001; Rötter et al., 2015). Boote et al. (2001) discussed several ways to use crop models to aid in plant breeding and remarked on the need for the improvement of models to describe cultivar-specific tolerances for drought, cold, heat, diseases, and pests. Rötter et al. (2015) reviewed the use of crop models in supporting ideotype breeding, providing several examples. Rincent et al. (2017) proposed a criterion to optimize multi-environment trials that combines crop simulation models and genomic selection models, which would result in more efficient evaluation of GEMI. Cooper et al. (2014) argued that future scaling of breeding programs would come from integration of germplasm knowledge, high-throughput genotyping and phenotyping, and modeling and prediction methods. Data acquisition, analysis, and prediction of performance of new genetic materials in multiple target environments will require tools such as remote and near-ground sensing, Internet of Things, cloud networking, algorithms and models, artificial intelligence, and other emerging technologies to assist rapid plant selection.

There has been increasing interest in combining crop and genetic simulation models. It has been proposed that plant breeding can be assisted by linking gene expression to traits that can be modeled, with the latter serving as input parameters of models to evaluate the performance of potential cultivars in multiple environments (e.g., Hammer et al., 2002; White and Hoogenboom, 2003). Cooper et al. (2014) reviewed the topic and present models as a component of the breeding strategy. Among several examples, Chapman et al. (2003) illustrated the use of models to evaluate genotype performance across multiple environments based on 15 genes controlling four adaptive traits and a quantitative genetic model simulating near isogenic lines for different combinations of traits. Messina et al. (2018) discussed the integration of crop models with whole genome prediction methodologies, which are applied in breeding to enable prediction of traits for new genotypes. These constitute the most advanced efforts in this area and provide a useful blueprint for modelers interested in integrating modeling with breeding. The integration of crop models with whole genome prediction is expected to open the potential for prediction of GEMI for breeding and product placement and for increasing the size of plant breeding programs without expanding expensive field testing (Technow et al., 2015; Messina et al., 2018). From a different perspective, Araus et al. (2018) reviewed strategies for improving and translating high-throughput phenotyping into genetic gain, including the use of crop models. To meet these expectations, the degree of detail and complexity of the processes represented in crop models and their performance require careful debate (Messina et al., 2009).

The success at making crop models useful for the assessment of GEMI depends on the effectiveness of modelers, breeders, and agronomists working together. But in any case, it is important that modelers assess model capabilities and input data quality realistically.

## Performance of Crop Models in Recent Evaluations

In recent years, under the umbrella of Agricultural Model Inter-comparison and Improvement Project (AgMIP), crop modelers have engaged in studies to evaluate model performance and provide avenues for the improvement of models (Ruane et al., 2017). Multi-model comparisons have been conducted for major staple crops, including wheat (Asseng et al., 2013), maize (Bassu et al., 2014), rice (Li et al., 2015), and potato (Fleisher et al., 2017). The standard approach has been to calibrate many models in selected world sites with increasing level of experimental observations made available to modelers. Even when complete calibration information is available to all modeling teams, important variation compared to observations and among models has been found. For example, Bassu et al. (2014) compared 23 maize models in four locations representing a wide range of maize production conditions (Lusignan, France; Ames, USA; Rio Verde, Brazil; and Morogoro, Tanzania), with individual models differing considerably in yield simulation at the four sites (2–4 Mg/ha for the 25 and 75 percentile with low level of information for calibration, and around 1 Mg/ha with high level of information). Similarly, Asseng et al. (2013) compared 27 wheat models at four sites (the Netherlands, Argentina, Australia, and India), obtaining a large variation in simulated grain yields when limited information was provided for calibration. After full calibration, the variation among models was reduced, and many models (>50%) simulated yields with uncertainties within 14% of the mean coefficient of variation found in over 300 wheat field experiments, indicating that model calibration and the choice of models for use in particular applications are important factors. A comparison of 13 rice models with multi-year yields obtained experimentally at four locations (Los Baños, Philippines; Ludhiana, India; Nanjing, China; and Shizukuishi, Japan) resulted in yield predictions by individual models differing by as much as twofold when low levels of information were provided for calibration (Li et al., 2015). When more complete calibration information was provided, the model variation was reduced, but no single model consistently provided reliable predictions of yield across sites and years.

Evaluations of model performance against experimental data [as by Basso et al. (2016) and Gaydon et al. (2017)] are steps in the right direction toward model improvement, as they may help uncover deficiencies. Based on the review of 215 papers including data from 43 countries, Basso et al. (2016) reported normalized RMSE of ~10, ~20, and ~10% for yield of maize, wheat, and rice, respectively, across all testing conditions. Better and worst performances were reported for individual cases, and for grain yield components and other variables. Gaydon et al. (2017) evaluation included 12 countries and diverse environments, crops, and management practices. They reported RMSE of 1,084 kg/ha for the combined rice data sets compared with the standard deviation (SD) among the observed data and replicates of 2,038 kg/ha. Similarly, RMSE and SD of 845 and 1,794 kg/ha for wheat and 1,004 and 2,408 kg/ha were reported. Gaydon et al. (2017) argued that the performance of a model is adequate if it can simulate the observed behavior within the bounds of experimental uncertainty. They also pointed out that good model performance requires overcoming significant challenges in the estimation of input parameters that may indicate deficiencies and the need for model improvement. The problem with these assessments is that they coalesce individual evaluations into broad-scope statistics that obscure many details or less than stellar performance. For example, Figure 2 in Gaydon et al. (2017) depicts a reasonable overall prediction trend including 326 pairs of simulated and observed wheat yields across diverse environments. However, the large departure from the 1:1 line of many pairs of points should give us pause if we consider the need for accurate assessment of the performance of genotypes in diverse environments.

The variation among model simulation results further increases when comparing projections in response to changing climate scenarios, including warming and elevated atmospheric CO_2_ (Asseng et al., 2013; Bassu et al., 2014; Li et al., 2015). In these comparisons, the variation among crop model outputs increases as temperature and CO_2_ move further from current conditions and represent a greater proportion of the uncertainty in climate change impact projections than variations among general circulation models (e.g., Asseng et al., 2013). These results indicate the need to improve crop models and can be interpreted as a warning call of their limitations for more demanding GEMI assessments. Understanding the underlying causes of such variations and identification of the best approaches to model individual processes, rather than just trusting the average, will speed up progress.

Multi-model comparisons have also demonstrated that the use of model ensembles based on the mean or median of all model outputs improves predictions. Bassu et al. (2014) reported close agreement between the mean of observed and the mean of simulated maize grain yields in the four locations used for evaluation, and this good agreement was obtained both with low and high levels of information available for calibration. The mean of an ensemble of rice models resulted in grain yield prediction uncertainty of about 5% of measurements across four locations, while no single model provided predictions with uncertainties of <10%. Asseng et al. (2013) and Fleisher et al. (2017) reported similar results.

Although for certain conditions, multi-model ensembles might be better than relying on individual model simulations for projecting future crop yields, Carter (2013) pointed out that finding the minimum number of required models is not simple, and as indicated by Wallach et al. (2018), multi-model ensembles are not a substitute to model improvement. Multi-model ensembles, which paraphrasing Quételet (Eknoyan, 2008) put their faith in “l’modèle moyen,” might be comforting as a means of reducing uncertainty in some applications, but their use is challenging or impractical for the routine application of crop models to evaluate GEMI. Just considering the scientist-time invested in multi-model comparisons for relatively simple cases should make that point clear.

## Where Are Model Improvements Required?

We focus on the major components of crop development and growth within crop models: phenology, which determines which resources the crop will access and to which stresses it might be exposed; solar radiation interception, which is determined by the green canopy development and its architecture and by the progression of senescence; water and nitrogen capture and use, which is determined by soil and root properties; net photosynthesis and biomass gain, which is determined by plant properties and limitations imposed by the environment; and biomass partitioning, which determines allocation of carbon and other elements to aboveground, belowground, and harvestable portions of the plant. Estimating the potential biomass production in a location is relatively simple when based on climate forcing. Once a suitable growing season length is defined, the available radiation, temperature, and dryness of the atmosphere bound the potential production of biomass. Most of the difficulties in modeling biomass production and yield with accuracy arise from defining the actual patterns of radiation interception, the effective soil volume explored by the roots, the interactions among stresses, and the switches or threshold-like responses that determine pollination failures or abortion. In what follows, we review the modeling components that determine potential growth and limitations based on resource capture, use efficiency as well as the definition of the sink size.

### Phenology

Crop growth simulation requires prediction of the timing of significant growth stages. These predictions are mostly based on thermal time accumulation modulated by photoperiod and in some cases vernalization. Models represent phenology satisfactorily (e.g., Aslam et al., 2017; Gaydon et al., 2017) mostly when calibrations and use are local, but are far from accurate even for crops with a wealth of information like maize (Kumudini et al., 2014) or winter wheat and after systematic careful calibration (Ceglar et al., 2019).

The calibration procedure also matters. Wallach et al. (2019) evaluated the prediction skill of the phenology components of 27 wheat models with special attention to the role of calibration. The data were from two check varieties in multi-year trials at multiple locations across France. The authors concluded that, overall, the models provided good predictions, with the median of mean absolute error of 6.1 days. Calibration compensated to some extent for differences between modeling approaches, while different calibration approaches caused differences in prediction error between similar modeling approaches.

Success in predicting relatively coarse patterns of development but difficulties obtaining accurate predictions when outside the calibration domain should hardly be a surprise. Slafer and Rawson (1994) stated in a thorough review that the controls of phenology in wheat are complex and subject to a degree of GxE that makes modeling and forecasting challenging. Our understanding of the controls of phenology has increased considerably. For example, Legris et al. (2016) have shown that phytochrome B is not only related to photoperiod but also to temperature sensitivity. Baumont et al. (2019) relate leaf appearance rate with carbohydrate availability and claim that the photoperiod effect of leaf appearance rate could be a surrogate for carbohydrate availability. And one could think that as our knowledge of the gene network controlling phenology improves, models will improve as well; but will models accelerate the uncovering of these networks? Models can help identify ideal development patterns for a given location: e.g., flowering early enough to escape heat and water stress but late enough to escape a late frost (e.g., Hunt et al., 2019), but it can be more difficult to assess GEMI beyond these broad brushstrokes.

### Canopy Development

Correct modeling of the canopy leaf area and architecture is essential for modeling solar radiation interception, and therefore crop growth and water use as well as soil shading (affecting soil water evaporation). The canopy architecture, the prevailing angle of the leaves within the canopy, modulates radiation interception and the distribution of radiation among the canopy elements. Defining the canopy greenness throughout the growth cycle is critical to compute transpiring (green area) and non-transpiring fractions of the canopy. Leaf development is largely a function of temperature and carbohydrates availability (Baumont et al., 2019), but leaf expansion is also controlled by water and nutrient stress.

Many models develop leaf area by simulating leaf appearance rate as a function of thermal time, and leaf expansion as a function of temperature and water and nitrogen status. In single stems of determinate crops such as wheat, leaf expansion ends near anthesis. Senescence of individual leaf segments may begin before anthesis and continues from anthesis to maturity. Thorough evaluations of canopy development simulations are scarce. Yoshida et al. (2007) evaluated model parameterization approaches to simulate leaf area development of nine rice genotypes grown under diverse environments. The different approaches resulted in relative root mean square deviation (normalized between 0 and 1) from 0.16 to 0.21 during calibration, and from 0.18 to 0.33 during evaluation with an independent data set. A comparison of 29 maize models resulted in large simulation departures from measurements of maximum leaf area index (LAI) in 8 years of measurements (Kimball et al., 2019). Cammarano et al. (2016) comparison of 16 wheat simulation models for four world locations shows large differences of simulated LAI between models and in comparisons with measurements (for example, maximum LAI twentieth and eightieth percentiles of 2-5 m^2^ leaf m^−2^ ground in Australia).

The fraction of the assimilated carbon (usually treated as biomass) that is apportioned to leaves is calculated through different means, all of which are empiric and are based directly or indirectly on phenology. Villalobos et al. (1996) followed a matrix partitioning approach for sunflower, where the fraction apportioned to leaves decreases in three steps from emergence to beginning of flowering, when it becomes zero. Jones and Kiniry (1986) and Hammer et al. (2009) calculated this fraction (biomass basis) in maize and sorghum, respectively, using the number of fully extended internodes as the basis for partitioning biomass to leaves (at 10 internodes, the fraction is ≈0.5), but there is significant dispersion in the regression (Figure 5 in Hammer et al., 2009). This approach has some semblance to that of the functional-structural model of Drouet and Pagès (2003), and provides a continuous change in the partitioning coefficient compared with the phasic approach in sunflower. Stöckle et al. (2003) followed an allometric approach, tying the partitioning of biomass to leaves to the biomass accrual per unit area. Fortunately, the largest impact of deviations in leaf area simulation occur when the leaf area index is lower than 3 m^2^ leaf m^−2^ ground, for beyond this threshold further increases in LAI cause proportionally smaller errors in radiation interception (unless the row structure is too sharp and “hedgerow” models are needed). Yet, connecting these parameters with the gene network controlling the processes defining leaf growth and development (Lastdrager et al., 2014) is still a challenge. Coarse phenology-based or allometry-based approaches are far from this level of detail. Understanding and modeling biomass allocation is likely one the areas that requires the most research and a better theoretical framework.

Large departures in canopy development can introduce uncertainties in other crop growth and resource capture processes and vice versa. While, the relatively simple models currently in use can provide a satisfactory stratum to test how changes in other processes affect the ultimate determination of yield, the network of genes that determine any process would at some point intersect the network of processes directing leaf development and expansion in greater detail. This is exemplified by the relationship between stem length and grain size (Miralles and Slafer, 1995); but how many of the less obvious linkages remain undetected? There is a risk in confusing a well-calibrated model with a model able to represent the level of detail in complex gene networks that are not even completely known, for example to model ABA-induced stomatal closure (Albert et al., 2017).

### Biomass Production

Mechanistic models of photosynthesis simulate gross photosynthesis and subtract growth and maintenance respiration to calculate net carbon assimilation. Carbon is partitioned into aerial (stems, leaves, and grains) and root portions, and expressed as biomass based on its carbon requirement and chemical composition, which are associated with growth and maintenance respiration (Penning de Vries et al., 1983). An advantage of these models is that photosynthesis and transpiration are linked *via* stomatal conductance, the latter responding to environmental conditions such as light, CO_2_ concentration, and humidity (e.g., Kremer et al., 2008). These models provide excellent explanatory frameworks, but their usefulness may be challenged by the large number of parameters, the correlation among parameters, uncertainties associated with their values, the need to integrate photosynthesis and transpiration throughout the crop canopy, and the growth-photosynthesis feedback.

Simulation of biomass production as a function of daily crop intercepted solar radiation multiplied by a conversion factor to biomass (*e* = radiation-use efficiency, g MJ^−1^) as defined by Monteith (1995) simplifies the prediction of crop biomass gain. This framework has been adopted by many modeling teams. The value of *e* can be determined in field experiments (Sinclair and Muchow, 1999; Stöckle and Kemanian, 2009) and while there is a general consensus on the maximum attainable *e*, for example for C_3_ and C_4_ cereals, there are studies often reporting *e* that can be 20% or more higher than somewhat accepted high values. Kukal and Irmak (2020) have reported maize *e* of 4.8 and 5.1 g/MJ of intercepted PAR, while the review by Stöckle and Kemanian (2009) reported a maximum *e* of 4 g/MJ (converting solar‐ to PAR-based *e*). Without unwarranted dogmatism, it is hard to operate when supposedly conservative scalars are assumed or accepted to vary to such extent.

The large differences in *e* for different locations and environments in which the soils would not suggest water stress as a limiting factor have been mainly associated to difference in the vapor pressure deficit of the air (*D*, kPa). Stöckle and Kiniry (1990) summarized *e* data for maize across diverse world locations, and found that *e* fluctuating from 2.9 to 4.4 g/MJ PAR was negatively correlated to *D*. This relationship was further supported by Kiniry et al. (1998), who pooled additional data for maize and sorghum, by Manrique et al. (1991) in potatoes, and by Kemanian et al. (2004) in wheat and barley. The main reason for such a response is that, as *D* increases and transpiration increases, stomata close (Monteith, 1995). It is difficult to separate diffuse radiation from *D* effects (Stöckle and Kemanian, 2009). Most of the sources of *e* variations are known (the same ones that affect photosynthesis), including environmental factors such as temperature, radiation and its distribution in the canopy, and air humidity, or by plant factors such as nutritional and water status, ontogeny, and source-sink regulation (e.g., Stöckle and Kemanian, 2009). However, the game of responses is seldom incorporated in crop models. There are conceptual similarities but important differences in a bottom up model that regulates stomatal conductance based on relative humidity (e.g., Collatz et al., 1991), lumped models that rely on *D* to define a maximum *e* (Williams, 1990), and models that use other controls over *e* (Villalobos et al., 1996).

Another approach to simulate biomass gain is based on the concept of transpiration-use efficiency (*w*), which is used in a limited number of models (e.g., Stöckle et al., 2003; Steduto et al., 2009). Good relationships between biomass gain (*B*) and transpiration (*Tr*) have often been reported, which improve by normalizing transpiration using climatic evaporative demand (e.g., de Wit, 1958) or *D* (e.g., Bierhuizen and Slatyer, 1965). Tanner (1981) and Tanner and Sinclair (1983) formalized this relationship deriving an expression accounting for the common stomatal pathway for carbon assimilation and water loss from crop canopies stating that *w* = *k*/*D*, where *k* is a crop/genotype parameter. This parameter was assumed constant for a given genotype, in large part because the ratio of internal (leaf) to external (air) CO_2_ concentration was assumed to be constant. Therefore, *B* = *w* × *Tr*. The value of *k* can be determined experimentally if *Tr* can be measured. However, the stomatal optimization hypothesis of Cowan and Farquhar (1977) states the marginal water use efficiency leans toward a constant; based on this assumption, it can be shown that *w* is proportional to the square root of *D* (*w* = *k*/*D^β^* with *β* = 0.5) and that the ratio of internal (leaf) to external (air) CO_2_ concentration decreases as stomata close. Kemanian et al. (2005) showed that this relation seems to hold true for many species and estimated that *β* = 0.59 for barley and wheat; Kremer et al. (2008) estimated that *β* = 0.44 for maize. Although the apparent alignment of theory and data is pleasing, there is substantial dispersion in any *k* estimation and variation among genotypes is hard to quantify and requires a refined understanding of the environmental interactions (Condon et al., 1993).

A shortcoming of the *e* approach is the decoupling between biomass production, the canopy energy balance, and the crop water use. The consequences of this decoupling can be exacerbated by deviations in simulation of crop water use discussed below. This occurs because biomass gain calculations based on *e* depend on intercepted PAR radiation, but do not consider the canopy energy balance, the soil-plant-atmosphere continuum, and ensuing changes in stomatal conductance. The consequences of this decoupling are significant (e.g., Basso and Ritchie, 2018). In the model CropSyst (Stöckle et al., 2003), this is resolved, at least for the growth estimation, by using the minimum of the growth estimated derived from radiation or transpiration.

Both *e* and *k* are, to some extent, negatively correlated. High *e* under low *D* would reflect a high stomatal conductance and high *k* may reflect lower stomatal conductance and therefore high *w*. These parameters, if used in combination, can be helpful to discern if aggressive water use (high *e* and high *Tr*) should be favored over a conservative use of water (high *k* and low *Tr*). Once again, these macro approaches can be robust enough (if well used) to simulate growth and can help define stress environments, and with expert use can suffice to explore the biological boundaries to growth. Beyond this relatively simple step, the task of evaluating the potential to genetically manipulating the expression of these traits belongs to more detailed photosynthesis models (e.g., Kannan et al., 2019; Wu et al., 2019). In our opinion, the expert user of a detailed simulation models must have a profound understanding of simplified approaches that retain core explanatory power and shed peripheral processes.

### Crop Water Use

Comparative studies have uncovered a large variation in model simulation of crop water use (Cammarano et al., 2016; Kimball et al., 2019), which stems from the combination of several factors. For example, models use a variety of approaches to determine atmospheric evaporative demand, to be referred to as crop potential evapotranspiration (CPET). This accounts for the energy available to evaporate water, and the conductance for water vapor between the exchange surface and the atmosphere per unit of land area, driving crop transpiration (mostly through plant stomata), soil water evaporation, evaporation of water intercepted by crop canopy and residues, and snow sublimation. The most biophysically complete approach to calculate CPET is the Penman-Monteith evapotranspiration (P-M ET) equation (Allen et al., 1998), which has been shown to outperform several other approaches when compared with lysimetric observations (e.g., Allen, 1986; López-Urrea et al., 2006; Benli et al., 2010). The Penman-Monteith ET equation is based on the combination of the energy balance and vapor and heat transfer equations to estimate water fluxes of crop canopies modeled as a “big leaf”. The P-M ET equation is not a perfect approach to model the complexity of water and heat fluxes from cropped surfaces, particularly the assignment of resistances to canopy and soil surface contributions before canopy closure. Limitations of the application of the P-M ET equation to real canopies have been addressed with engineering approaches using empirical adjustments, mostly based on lysimetric data (Allen et al., 1998).

Other methods to approximate CPET fluxes have been developed based on increasing simplifications of the P-M ET equation to accommodate the use of weather data with less variables than required. However, each simplification deviates from the physical transparency of the P-M ET approach and forces incorporating empirical coefficients whose values are not easy to assess without careful calibration and still produce CPET estimates that depart from PM-ET. Kimball et al. (2019) highlight this problem. These authors compared potential ET from 29 maize models, reporting huge differences among them (Figure 10 in Kimball et al., 2019), which obviously propagated to the simulation of actual evapotranspiration, crop transpiration, and beyond.

There are also many models to simulate crop water uptake (normally equated to *Tr*), including a wide range of complexity (e.g., van den Berg et al., 2002; Wang and Smith, 2004; Camargo and Kemanian, 2016). Evaluation of the performance of these models or sub-models decoupled from complete crop models often reveals important differences that can be obscured when comparing aggregated variables like yield. Camargo and Kemanian (2016) compared the water uptake methods implemented in six crop models, ranging from simple empirical to more mechanistic approaches, in scenarios with different evaporative demand, soil texture, and water distribution with depth. They found that each method responded differently to these scenarios, affecting the onset of water stress, the cumulative water uptake, the shape of the soil drying front, and the response to high transpiration demand. If root depth progression and water uptake were genotype-agnostic, then crop models could be calibrated and used for GEMI analysis of other traits without much concern for the roots. But we know that is not the case, and the interaction of the type of model used for modeling root colonization of the soil profile and algorithms to simulate water uptake are of critical importance in any analysis, and more so for GEMI assessment which demands a fine slicing of differences among genotypes.

Uncertainty in the calculation of potential *Tr* and realized crop water uptake is compounded by two-way feedbacks with canopy and root growth, affecting biomass growth and yield projections. Cammarano et al. (2016) quantified variations among 16 wheat models in the simulation of actual evapotranspiration, water use efficiency, transpiration efficiency, crop transpiration, soil water evaporation, and grain yield at increased temperature and elevated atmospheric CO_2_ concentration. The uncertainties in the simulation of evapotranspiration and *Tr* were greater with high temperatures and in combination with elevated CO_2_. They concluded that the simulation of crop water use should be improved and evaluated with field measurements before models can be used to project future crop water demand (Cammarano et al., 2016). The logical follow up question is what to do next. Is it really the case that models need to be improved to simulate water use? Perhaps soil input information needs to improve, and the consideration of plant-soil interactions needs to improve, but modeling approaches should converge to those with a defensible theoretical and empirical basis.

### Crop Nitrogen Use

The N content in plants is typically modeled in two steps: (a) crop N demand and (b) soil/root N supply, with the minimum of the two reflecting crop N uptake (Stöckle et al., 1994, 2003). Above ground N demand is often calculated based on three standard N concentration curves evolving daily as a function of aboveground biomass (Greenwood et al., 1990; Stöckle and Debaeke, 1997): maximum (upper limit), critical (below which growth begin to be affected), and minimum (growth stops). The daily crop N concentration and biomass growth reduction, if any, is defined by soil N supply. These standard concentration curves are determined from experiments including different levels of fertilization (Stöckle and Debaeke, 1997). A model by Jamieson and Semenov (2000) simulates N demand separately for structural N (low N concentration), green area N (high N concentration), and storage (luxury N consumption), not requiring the standard curves. In either case, the amount of N apportioned to roots must be calculated. The N supply is simulated based on the N mass, root density, and soil water content of all soil layers explored by roots. The supply of N is reduced as soil N mass and water content decrease from optimum values, eventually not meeting N demand and affecting leaf area expansion and radiation-use efficiency. Both demand and supply processes in crop models are empirical and potentially subject to large uncertainties.

In the case of wheat and other grain cereals, N accumulation in grains and projection of protein content are important. Most models are limited to the prediction of N concentration, which is converted to a protein content, although models that simulate the content of storage proteins are also available (Martre et al., 2006). A robust allometric relationship between grain N concentration, harvest index, and N concentration in aboveground biomass at harvest was shown by Kemanian et al. (2007a) for maize, sorghum, wheat, and barley, which indicates that the timing of N uptake and biomass accretion has lesser influence on the correlation between final C and N partitioning to grain.

Implementing different approaches to represent crop N use results in substantial diversity when outputs from different models are compared. A comparison of three spring wheat models in Canada showed that all models provided good predictions for average plant N when precipitation was near normal and recommended N rates were applied, but performance decreased when N was applied at lower rates or in the presence of mild precipitation deficit or excess early in the season (Sansoulet et al., 2014). A comprehensive study evaluated 11 crop models for spring barley in Jokioinen, Finland, under different N fertilization rates (Salo et al., 2016). The models differed widely in process description. Although detailed data were provided for calibration, the authors showed that model performance decreased for N-limited conditions and when environmental conditions deviated strongly from the calibration conditions.

Models provide opportunities to evaluate hypotheses of plant N dynamics (e.g., Sinclair and Amir, 1992; Jamieson and Semenov 2000), but the use of models for GEMI evaluation faces challenges. Multi-model comparisons indicate large variations in model responses, indicative that some models may have inadequate representation of processes or/and unsatisfactory selection of input parameters by users. The problem gets compounded by hydrologic and soil processes affecting the movement of nitrate in the soil, and other processes affecting the soil C and N dynamics responsible for N mineralization and immobilization as well as N transformations.

### Yield

Grain yield is often modeled using yield components, which are affected by environmental factors. However, the ability of crop models to simulate grain number, grain weight, and translocation of stem reserves is often inadequate (e.g., Moreno-Sotomayor and Weiss, 2004; Dettori et al., 2011; Gaydon et al., 2017). Dettori et al. (2011) reported simulated grain yield with an average normalized root mean square error (nRMSE) of 27–20% compared with observed yields for three wheat cultivars and at two sites under Mediterranean conditions in Italy. Evaluation of a rice model simulations of grain yield based on 11 studies resulted in an average normalized RMSE of 23%, with two studies reporting a value of 3%, five in the range ~21–18%, and four in the range of 32–23% (Timsina and Humphreys, 2006). The same study reported eight studies for wheat, with normalized RMSE of simulated grain yields of 13%, and a range of 17–2%.

The prediction of grain yield is the result of numerous processes occurring during the growth cycle. Jamieson et al. (1998) concluded that for yield prediction the accurate simulation of phenological development and LAI is much more important than the components of the yield. Sinclair and Jamieson (2006) argued that the correlation between growth rate at a time before anthesis and grain numbers, and between the latter and grain yields led to models with unnecessary complexity. Under no N limitation, a mechanistic model of biomass accumulation and a harvest index for partitioning to grain accounted for most of the variability in wheat grain yield over a 10-year period (Amir and Sinclair, 1991). A similar argument underpins the Kemanian et al. (2007b) model to calculate the harvest index in determinate crops; the model has a logical foundation, a minimum number of parameters, but requires that phenology and growth be modeled accurately.

While it is tempting to argue that simple models are likely more robust than yield-component based models to predict yield, it is easy to see that these models can be of limited use when the interest is understanding the controls of the grain number and size and GEMI. Bustos et al. (2013) showed experimentally that high grain number can be combined with high grain weight in wheat, showing impressive yield gains in a high yielding environment. Interestingly, the high yields were associated with extremely high *e* compared with check wheat cultivars or any C_3_ crop and suggests a strong sink control over photosynthesis. To our knowledge, most models simulate first the photosynthesis rate (or the lumped surrogates *e* × *S_t_* or *w* × *T*) or how much biomass will be available for the growth of each organ, and do not include a feedback from growth potential to photosynthesis, a feedback that in any case is challenging to model.

Another factor introducing uncertainty in grain yield prediction is the effect of drought and cold and heat stress on grain set and growth. We are specifically referencing to effects that are independent or in addition to the impact of these stresses on photosynthesis. Prasad et al. (2017) reviewed the effect of short episodes of heat stress on 20 field crops, showing reduction of grain set and harvest index with temperatures above crop-specific thresholds, ≈32°C in the case of wheat. The ability of crop models to simulate grain yield under conditions of heat stress appears constrained, which can be a significant limitation considering global warming scenarios. Liu et al. (2016) compared four wheat models with 4 years of environment-controlled phytotron data with two cultivars under heat stress, concluding that all models needed improvements in simulating heat stress during anthesis. Schlenker and Roberts (2009) and Hoffman et al. (2020) analyses of large panel of county level yields reveal threshold-like responses to temperature for maize, sorghum, soybean, and cotton. Furthermore, maize and soybean seem to have broad plateaus in which temperature has a moderate effect on yield, while sorghum has a more sensitive response with almost no plateau that is more sensitive in the cold end but slightly more adapted in the hot end of the data domain (Hoffman et al., 2020). It is not clear if current models represent these nuances with fidelity.

### Roots and Soils

The root-soil complex is likely one of the most understudied components and one that is represented with simplicity in simulation models. Much like foliage development, the root exploration of the soil volume depends on intrinsic properties of a given genotype that define the 3D structure of the root system, i.e., the progression of the rooting front and proliferation of roots in the explored volume, and the feedback response to soil properties that may limit or promote root growth. Roots need to intercept water or nutrients that can move through the medium or need to reach water or nutrients that are moving slowly through the soil. Lynch and collaborators (e.g., Lynch, 2011) have performed some of the most fundamental work on root architecture and its relationship with phosphorous (P) and N capture. This body of work shows that significant differences among genotypes within a species exist in root architecture and nutrient acquisition. Structural-functional models of root systems have been incorporated in models that, however, do not simulate full crop cycles (Schneider and Lynch, 2018), are not integrated in comprehensive crop models, and like any model carry the risk of conflating model assumptions with emergent properties. These models are far from representing the uneven exploration of soil layers by roots and the impact of compacted soil layers on actual water use patterns (Breslauer et al., 2020). New efforts at mechanistically modeling water uptake in soil layers with clusters of roots (uneven root distribution) are fortunately emerging in the literature (Graefe et al., 2019).

Most importantly, these models have little feedback from soil properties that may limit root growth. Ernst et al. (2016), working with wheat, and Stefani-Fae et al. (2020), working with soybean, have shown that crop yield responds strongly to soil physical properties that are best related to field measured soil hydraulic conductivity. Field measured saturated hydraulic conductivity can exceed that obtained from pedotransfer functions usually used in models by a factor of 10–20 (Stefani-Fae et al., 2020). To our knowledge, no model can yet represent this response mechanistically or derive these responses just by looking at a soil description. In the assessments of GEMI in conditions in which the yield variation is dominated by soil factors other than depth to bedrock, there are plenty of opportunities for uncertainties in root-soil processes to override genotypic variation as represented in crop models. These concerns are of lesser importance in irrigated and well-fertilized crops but become more important as soil limitations become more relevant. It is plausible that using remote sensing and machine learning algorithms (Azzari et al., 2017) to support earlier efforts at model inversion (Paz et al., 1998) can mitigate some of the soil-derived uncertainty, but clearly there is a long distance to travel to make models useful for GEMI assessments while at the same time assuaging concerns about uncertainty derived from soil variation.

## The Larger Context For Improving and Applying Models For Gemi Assessments

Crop models can potentially be useful for GEMI assessment, although uncertainty in output results will always exist depending on the specific model and growth conditions, and as reviewed above, with many possible interacting factors. Therefore, it is important for users (and developers) to carefully evaluate the context in which the models are applied as well as to be mindful of areas of model limitations. The context for improving and applying models includes the nature of the models themselves and appropriate knowledge of the environmental and management conditions under which crop model simulations are conducted.

### The Nature of Crop Models and Their Use

Crop models are often not well balanced in the treatment of the large number of processes and interactions that are needed. This usually reflects the modeling team composition, which leans toward more emphasis on quality and details of the mathematical formulation of processes in their areas of expertise, while other components are (much) less developed. Cooperation between modeling teams would be highly desirable for progress toward better models, including sharing of code and concepts and continued testing of models. Studying what has been done before embarking in large modeling undertakings seems to be of critical importance to accelerate innovation. Increased cooperation has been fostered by communities of crop modelers such as AgMIP (Ruane et al., 2017), MACSUR (Ma et al., 2014), and others. Activities in these communities have mostly focused on model comparison, with the shortcoming that emphasis has been placed on the performance of complete crop (“branded”) models, and much less on processes. The large diversity of model outputs in these comparisons (reviewed above) and underlying causes are difficult to identify, with multiple interactions and error propagation among different components defying quantification. The lack of experiments purposely designed to produce data for process comparisons is a barrier. Nevertheless, even comparison of individual processes using prescribed weather/soil scenarios and state variables affecting the target process would be extremely useful. This would require the selection of different approaches used in crop models, and coding them into a common platform for comparison (Jara and Stöckle, 1999; Camargo and Kemanian, 2016).

Another factor affecting the performance of crop models, often ignored, is the proficiency of model users. Choosing model parameters requires an understanding of the crops and environments involved, and knowledge of the model structure, processes, mathematical formulations, and sensitivity of model responses to changes in parameter values. Confalonieri et al. (2016) argued that one should not speak of evaluation of a model but rather of a model-user combination, where a major role of the user is in determining the method of calibration and the selection of crop input parameter values. This was explicitly shown in model simulations of crop evapotranspiration (Kimball et al., 2019), with very different results obtained by the same models operated by different users.

One barrier for judicious evaluation of potential model limitations is that model descriptions are often incomplete or lacking sufficient detail. Furthermore, model developers are continuously adding new capabilities and expanding their portfolio of projects in response to ever-growing demand for new applications from multiple users. However, if attention to the basic issues discussed in this article do not receive sufficient priority, crop models run the risk of losing credibility and relevancy.

### The Model Application Landscape

Successful model-based assessment of GEMI not only require the use of the better crop models, but an adequate representation of the environmental conditions on the landscape where crops are grown, and good knowledge of the management practices used. For regional or basin-scale assessments, the information on management practices is normally insufficient, starting with such simple facts as the temporal and spatial variation of planting dates. Similarly, weather and soil data are often inadequate, incomplete, or available at too large scales. Under these conditions, thorough crop model calibration is not always possible, and in fact the contrary is normally true. How do we calibrate models with imperfect information? Part of the answer is in the use of robust crop models whose state variables do not jump outside reasonable limits of variation under extreme or new conditions, as well as greater emphasis on crop input parameters that are observable.

Landscape topography, local and basin surface and sub-surface hydrology, presence of shallow water tables, field flooding, soils with physical or chemical challenges for roots colonization or crop growth, variations of carbon and nitrogen cycling, the effect of crop rotations, cover crops and residue management, and other factors are part of the landscape context where models must be applied for GEMI assessment. These are not trivial barriers that could be partially mitigated by hydrologic models, linking crop models with spatial models of water and nutrient transport, carbon and nitrogen cycling models, remote sensing data, and other tools. But these also have their own uncertainties and require expertise outside the interest of crop model users. An example, which is perhaps extreme, is the yield variation in the loess deposits of the Palouse region in Washington, Oregon and Idaho (Huggins et al., 2014). Because of the interaction of topography and landscapes, the soils represent almost contrasting climates. All these variations reflect not only in yield but also in nutrient dynamics and grain N concentration of the wheat and barley typically grown in this region. In this physical context, 1D models can be useful to represent trends but are relatively hopeless at capturing granular, topographically driven variation.

There is also an agronomic and biological context to consider. Not many models can simulate crop rotations, cover crops, and residue management. Crop models do not consider the large number of organisms and the continuously changing pressure from weeds, pests, and diseases; and if they do, properly capturing the biological variation and known responses to the environment of these bio-stressors is an additional challenge. Nonetheless, recent work with large data panels and machine learning (Schlenker and Roberts, 2009; Hoffman et al., 2020) indicate that a substantial fraction of the yield variation can be captured with relatively simple models. This indicates that some putatively complex interactions are not always relevant or that aggregation at certain scales (e.g., counties) dampens the expression of these interactions in the data.

## Final Remarks

This article represents a view from a crop modeler perspective looking into the progress needed to further model applications addressing GEMI. Depending on the type of application, some but not all models may perform reasonably well under well-constrained conditions. Both model and user performance often deteriorate when the simulated conditions depart from the calibration domain or typical testing scenarios. This challenge has been addressed for some applications using model ensembles. While model ensembles provide cover against model and sometimes input uncertainty, further progress needs to break free from ensembles to assess models’ weaknesses and knowledge gaps critically.

Modeling teams may focus on the following: (1) There must be convergence on how to model biophysical processes for which the basic understanding has been in place for decades. (2) While model development always demands sagacity to integrate principles and empirical knowledge, the space requiring the most work is likely the root-soil interaction to determine root exploration and water uptake as well as nutrient acquisition. Sometimes maximum rooting depth or root distributions are imposed without empirical support or calibrated with substantial supervision. Yet being able to predict rather than impose how roots explore the soil (or how much water is accessible) is of critical importance for practical applications. (3) Sewing trait expression and modeling to gene transcriptional and posttranscriptional controls will require a tight bottom-up and top-down coordination of models, and that requires teams with balanced expertise. This is difficult to accomplish. (4) Most crop models are 1-D, while many landscape processes depend on the interaction of topography and soil properties. This is one of the areas with the potential to truly exploit GEMI for a more refined management of the landscape. (5) Models cannot become even more difficult to use; setup, calibration, and application should be seamlessly integrated, otherwise the user may have more influence on the output than the model. (6) Data assimilation strategies that allow ingesting data at runtime and updating state variables while conserving mass and energy will be critical to integrate models to a flexible data-model. In this context, it is conceivable that the integration of sensors, artificial intelligence, and other technologies will be helpful to reduce uncertainty, but improvement remains a sine-qua-non condition for crop models success as research and applied tools.

The question “Can crop models identify critical gaps in genetics, environment, and management interactions?” has many angles, requiring careful work by multidisciplinary teams to overcome the limitations discussed in this article. The context for crop model applications is complex, requiring ingenuity, dedication, and good judgment to advance GEMI assessments and other applications.

## Author Contributions

Both authors contributed substantially to write this article.

### Conflict of Interest

The authors declare that the research was conducted in the absence of any commercial or financial relationships that could be construed as a potential conflict of interest.
